# 4489 Association between surgery with general anesthesia and cognitive decline in older adults: analysis using shared parameter models for informative dropout

**DOI:** 10.1017/cts.2020.168

**Published:** 2020-07-29

**Authors:** Phillip Schulte, Katrina Devick, Juraj Sprung

**Affiliations:** 1 Mayo Clinic

## Abstract

OBJECTIVES/GOALS: Recent studies have assessed the association between surgery with general anesthesia and cognitive decline in longitudinal cohorts of older adults. Patients diagnosed with dementia more frequently drop out of these longitudinal studies or are unable to complete the test battery. We revisit this aim with focus on methods for informative dropout. METHODS/STUDY POPULATION: We use data from the Mayo Clinic Study of Aging (MCSA), a longitudinal epidemiological study of the prevalence, incidence, and risk factors for mild cognitive impairment (MCI) and dementia. Our primary outcome of interest was global cognitive z-score, assessed at study visits every 15 months. We implement linear mixed effects models to assess the association between post-enrollment exposure to surgery/anesthesia and subsequent cognitive decline trajectories. Demented patients more frequently drop out of MCSA, so, subjects with the worst cognitive outcomes are unobserved and missing data may be informative. Since this missingness may be missing not at random, we use shared parameter models to analyze continuous cognitive outcomes while jointly modeling time to dementia. RESULTS/ANTICIPATED RESULTS: A total 1948 subjects, non-demented at baseline, from the MCSA were included. Median age was 79, 51% of subjects were male, and 16% had MCI at enrollment. Among median follow-up of 4 study visits over median 5.4 years, 172 patients developed dementia and dropped out from further assessments of cognitive function. In adjusted linear mixed effects models, our data suggest post-enrollment exposure to surgery/anesthesia is associated with a decline in cognitive function over time (change in slope = −0.07 standard deviations of cognitive z-score per year, 95%CI = −0.08, −0.05, p<.001). After adjusting for informative dropout using shared parameter models, surgery/anesthesia is associated with greater cognitive decline (change in slope = −0.14 per year, 95%CI = −0.16, −0.12, p<.001). DISCUSSION/SIGNIFICANCE OF IMPACT: We revisited a prior analysis by our group with consideration of informative dropout. Subjects who dropout due to dementia may have different trajectories of cognitive decline compared to non-demented subjects. Shared parameter models estimate the association between surgery/anesthesia and cognitive decline accounting for informative dropout.

